# Surgical Treatment in Hypertension
*From an address by Dr. Horace Evans to the Devon and Exeter Medico-Chirurgical Society on October 26th, 1948.


**Published:** 1948

**Authors:** Horace Evans

**Affiliations:** London


### SURGICAL TREATMENT IN HYPERTENSION *

BY

DR. HORACE EVANS, F.R.C.P.

*London.*

---

In opening his address the speaker reviewed the history of hypertensive disease. Richard Bright considered hypertension was secondary to nephritis, Sir Clifford Allbutt showed that in the majority of cases there is no clinical evidence to support that view: what he named "hyperpiesia" is now called benign essential hypertension.

Goldblatt produced hypertension in the dog by partially occluding one kidney and Wilson and Byrom repeated this experiment on rats and observed histological changes in the opposite kidney: if the clamped kidney was removed early hypertension did not develop. From this it was deduced that the kidney produces a pressor substance; but prolonged search for anti-pressor substances has proved fruitless. Trueta by stimulating the sympathetic produced cortical ischaemia of the kidney with resulting hypertension.

There is some difference of opinion as to what constitutes hypertension. The speaker considered that a systolic pressure of 180 mm. Hg. is certainly the upper limit of the normal range—he was inclined to put it lower: a persistent diastolic pressure of 100 mm. Hg. is pathological. But at a single examination a healthy young man may show pressures of 150/100. Women stand hypertension better than men. Benign essential hypertension constitutes 90 per cent. of all cases of hypertension. There are no symptoms (unless the patient is told he has "blood pressure"). Malignant hypertension is somewhat rare: the patients are usually men in the fifties—they look and feel ill and usually die within two years.

The medical treatment of hypertension—rest, cardiotherapy, potassium thiocyanate—is disappointing. Surgery has possibilities. Smithwick has devised the operation of bilateral dorso-lumbar sympathectomy. The speaker considered pedicle sympathectomy gave as good results with less risk. Operation has only a limited use. Those most likely to benefit are young patients with severe hypertension and retinitis; young women who, during pregnancy, develop hypertension which persists a year; and those with benign hypertension and high diastolic pressure. Operation should not be done on those over fifty. Certain unpleasant sequelae must be expected, such as sterility in the male.

Sympathectomy apparently cures retinitis and relieves other symptoms, especially headache. In a series of one hundred patients the operation had been worth while in most cases, but few had shown great improvement. Very few survived four years but they lived longer than would be expected without operation.

*From an address by Dr. Horace Evans to the Devon and Exeter Medico-Chirurgical Society on October 26th, 1948.

